# Knowledge attitude and convenience on self-medication practices among university students in Bangladesh exploration using structural equation modeling approach

**DOI:** 10.1038/s41598-024-60931-9

**Published:** 2024-05-12

**Authors:** Mortuja Mahamud Tohan, Faysal Ahmed, Israt Jahan Juie, Anamul Kabir, Md. Hasan Howlader, Md. Ashfikur Rahman

**Affiliations:** Development Studies Discipline, Social Science School, Khulna University, Khulna, 9208 Bangladesh

**Keywords:** Knowledge, Attitudes, Practice, Self-medication, Students, Bangladesh, Health care, Medical research, Risk factors

## Abstract

Self-medication is a prevalent practice among university students globally and is a significant public health concern. However, previous research has been limited in scope, focusing primarily on adolescents or the general public, leaving a gap in understanding the causal relationships associated with self-medication; thus, this study aimed to investigate the factors influencing self-medication practices among university students in Bangladesh by developing a comprehensive causal model. Data from 417 students across five public universities were collected using the simple random walk technique by a team of 10 members. The study utilized constructs of knowledge, attitude, and convenience related to self-medication as independent variables, while self-medication practice as the dependent variable. One-way ANOVA and structural equation modeling (SEM) were employed to develop a causal model of self-medication practice among university students in Bangladesh. The findings revealed that students with better medication knowledge and adverse drug reactions (ADRs) were more likely to practice self-medication. A positive attitude towards self-medication and ADRs was also significantly associated with higher self-medication practice scores. Additionally, those who perceived self-medication as convenient and prescribed medication as inconvenient had higher self-medication practice scores. The attitude towards self-medication had the most substantial negative effect on self-medication practice, followed by the inconvenience of prescribed medication and the convenience of self-medication. The model explained 87% of the variance in self-medication practice, indicating a good fit for the data. University students in Bangladesh possess intermediate knowledge of medication and primary knowledge of ADRs. They exhibit a positive attitude towards self-medication and ADRs. Physical convenience favors self-medication, while the inconvenience of prescribed medication contributes to its lower preference. Policymakers should focus on evidence-based guidelines to reduce the extent of unnecessary self-medication practice and to enhance the quantity and accessibility of prescribed medications to address the issue effectively.

## Introduction

Self-medication, defined as the practice of diagnosing and treating one's own illnesses without seeking professional medical advice, is a common practice among university students worldwide and is considered a significant public health concern^1–3^. While self-medication is considered a convenient way to address common health issues, it poses significant health risks, such as adverse drug reactions, drug interactions, and treatment failure^4–6^. Self-medication can also lead to antibiotic resistance, a global health challenge^7^. The misuse and ineffective utilization of medications are significant issues worldwide, with approximately 68% of European countries and 42% of American nations reporting such problems^4,8^. Both developed, and developing countries commonly engage in self-medication, although the prevalence is higher in developing nations^5^. The occurrence of self-medication in developing countries exhibits a wide range, spanning from 12.7 to 95%^9^. South Asian countries, in particular, demonstrate higher rates of self-medication. For instance, Nepal has a rate of 59%, Pakistan has 51%, and Bangladesh shows rates of 81.3% among the younger population and 78.5% among the elderly^10^.

If self-mediation is used responsibly, it can help conserve limited medical resources by addressing minor ailments, the burden on healthcare facilities, and reduce costs and time associated with seeking professional care for minor illnesses^11^. Due to that, WHO has pointed out that appropriate self-medication can be beneficial in treating acute ailments that do not require medical consultation. But it also has a flip side which can cause serious problems such as adverse drug reactions, treatment failure, antimicrobial resistance, etc. In Bangladesh, antimicrobial resistance is a significant issue, primarily attributed to unethical drug sales practices. A recent petition filed in the High Court of Bangladesh aimed to address this problem by seeking legal action against the immoral selling of drugs ^12^. It was highlighted that as much as 80% of deaths caused by infections were linked to antimicrobial resistance ^13^.

Although recently the healthcare system of Bangladesh has expanded its coverage and infrastructure has also improved substantially still it is considered suffering a budget shortage as only 5.4% of the GDP is allocated for its national healthcare system^14^. Consequently, there is a lack of accessible health insurance, both at the national and private levels^15^. In addition, the prevalence of self-medication in the country is exacerbated by the presence of unethical drug sellers, inadequate regulatory measures, and the widespread availability of prescription drugs without the requirement of a prescription^16^. These factors collectively contribute to the high prevalence of self-medication practices in Bangladesh. University students, particularly in developing countries, are highly susceptible to self-medication due to influential factors such as media and internet exposure promoting this behavior. Additionally, factors such as low perception of associated risks, easy access to the internet, increased pharmaceutical advertising without regulation, availability of drugs, level of education, and social status contribute to this trend^17,18^. Previous studies have reported high prevalence rates of self-medication among university students, including rates of approximately 76% in Karachi, Pakistan^17^, 87% in India^19^ 98% in Palestine^20^, and 43.2% in Ethiopia^21^.

Among university students, the prevalence of self-medication is notably high^22^. However, irresponsible self-medication can lead to various negative consequences. It is crucial to identify the influences on self-medication practices among university students, considering that they are generally knowledgeable about medication yet still susceptible to engaging in high levels of self-medication. Mohamed Elkalmi et al.^23^ highlighted that 77.2% of female students exhibit a preference for self-medication over prescribed medication due to their reluctance to venture out^24^. This sentiment was echoed by Raut et al.^25^, who observed that females, especially those experiencing menstrual problems, tend to practice self-medication more frequently^26^. Conversely, Parihar et al.^27^ found that the rate of self-medication is higher among male medical students^28^, with Mehta and Sharma^29^ identifying no significant disparity between genders^30^.

Moreover, Parihar et al.^27^ revealed that while most university students possess prior knowledge about self-medication, they opt for this approach primarily to avoid visiting doctors for minor ailments^28^. Convenience and the hope of prompt relief were cited as additional motivators for self-medication. Contrarily, Beyene et al.^31^ argued that self-medication is most crucial in emergency situations^32^. However, Parihar et al.^27^ and Johny et al.^33^ cautioned against the risks associated with self-medication, including adverse drug reactions (ADRs), drug dependence, and misuse of medication^28,34^. In terms of students' understanding of medication, Raut et al.^25^ noted that a majority of university students possess adequate knowledge about medications, often acquired through online research^26^. However, a notable proportion (16%) lacks understanding or any knowledge about medication. This underscores the importance of education and awareness campaigns regarding responsible medication use among students.

Johny et al.^33^ highlighted that many students are confident in their ability to manage minor illnesses independently^34^. Conversely, Mohamed Elkalmi et al.^23^ revealed a perspective among students that self-medication is considered part of self-care and is perceived as harmless^24^. However, Beyene et al.^31^ cautioned that without proper knowledge, self-medication can pose serious risks and consequences^32^. In terms of common health issues prompting self-medication, findings vary among studies. Raut et al.^25^ identified cough, diarrhea, and acidity as prevalent issues for which students practiced self-medication^26^, while Beyene et al.^31^ and Mohamed Elkalmi et al.^23^ cited fever and headache as primary reasons^24,32^. Parihar et al.^27^ expanded the list to include symptoms such as gastric pain, constipation, vomiting, and the common cold^28^. Furthermore, Johny et al.^33^ reported that analgesics, antipyretics, paracetamol, and ibuprofen were commonly used for self-medication^34^, with Parihar et al.^27^ adding antibiotics, antihistamines, antianxiety, and antifungal medications to the list^28^.

Regarding the frequency of self-medication, Raut et al.^25^ noted that many students occasionally practice self-medication^26^, while Johny et al.^33^ found that on average, students engage in self-medication 1–5 times within a year^34^. Previous research has identified several factors contributing to self-medication practices, such as knowledge and literacy^24,26^, attitude^28,30^, and the convenience of self-medication^32,34^ were identified as the most significant contributor to self-medication practices. However, how these variables specifically affect university students nationwide remains uncertain. Additionally, the inconvenience of prescribed medication (e.g., high cost, unavailability) in Bangladesh may also play a distinct role in self-medication practices among university students^15,16^. Previous studies in Bangladesh have primarily focused on self-medication among medical or medical-related students^9,35,36^ or the general public^13,37^ lacking a comprehensive understanding of the causal relationships among self-medication practices and other factors. Addressing this research gap, this study aims to investigate the factors (knowledge, attitudes, and convenience) influencing self-medication practices among university students in Bangladesh by developing a causal model.

Factors such as knowledge and literacy^24,26^, attitude^28,30^, and the convenience of self-medication^32,34^ have a significant impact on self-medication practices. However, how these variables specifically affect university students nationwide remains uncertain. Additionally, the inconvenience of prescribed medication (e.g., high cost, unavailability) in Bangladesh may also play a distinct role in self-medication practices among university students^15,16^. Previous studies in Bangladesh have primarily focused on self-medication among medical or medical-related students^9,35,36^ or the general public^13,37^ lacking a comprehensive understanding of the causal relationships among self-medication practices and other factors. Addressing this research gap, this study aims to investigate the factors (knowledge, attitudes, and convenience) influencing self-medication practices among university students in Bangladesh by developing a causal model.

### Theoretical framework

Self-medication has been a widely acknowledged phenomenon, with numerous attempts made to identify the factors influencing this practice. The Knowledge, Attitude, and Practice (KAP) model is commonly employed in this context, positing that existing knowledge shapes a favorable attitude toward self-medication, which in turn enhances the practice^38–40^. Studies such as those by Alves et al.^41^ underscore the role of knowledge in promoting safe self-medication practices, preventing indiscriminate use . Shitindi et al.^42^ contend that a lack of proper knowledge about medicines is a strong predictor of self-medication practices. Conversely, research by Abebe et al.^2^ suggests that while university students may possess good knowledge about self-medication, negative attitudes towards it are associated with reduced practice. Alkhawaldeh et al.^43^ and Alves et al.^41^ further supports this, noting a correlation between negative attitudes and reduced self-medication.

A critical aspect identified in various studies is the knowledge about adverse drug reactions (ADR) and the attitudes towards them, which significantly influence self-medication^42,44^. Studies by Gedam and Kuchya^45^ and Sivadasan et al.^46^ emphasize the importance of knowledge about ADR in shaping proper attitudes and responsible self-medication practices. Consequently, understanding ADR knowledge and attitudes becomes imperative for comprehensive research on self-medication.

Beyond the KAP model, the convenience of self-medication, juxtaposed with the inconvenience of obtaining prescribed medication, emerges as another influential dimension. James and French^47^ highlights that individual perceive self-medication as time-saving, economical, and providing quick relief. Afzal et al.^48^ argue that convenience is often driven by factors such as easy drug availability, self-confidence, and lower socioeconomic status. In countries like Bangladesh, there is a high prevalence of over-the-counter drug selling^49^, enhancing the convenience of self-medication. Conversely, obtaining prescribed medication in Bangladesh can be challenging, requiring considerable time and money, particularly for university students^50,51^. This inconvenience may significantly impact the actual practice of self-medication among university students, emphasizing the need to consider convenience as a crucial dimension in understanding predictors of self-medication.

**Figure 1:**
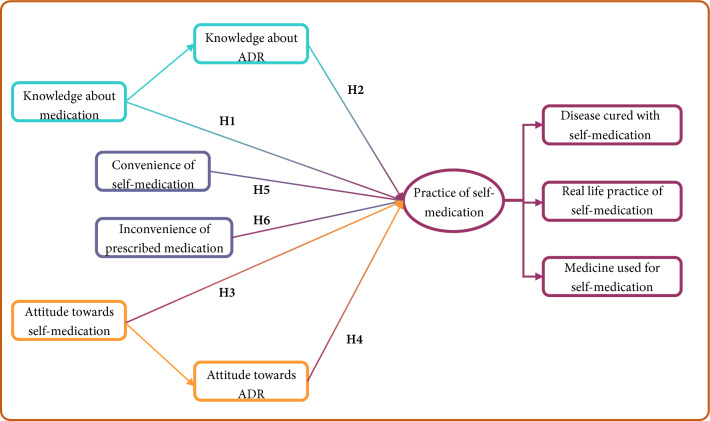
Conceptualized hypothesis.

Therefore, this study proposes following hypothesis (Fig. 1):

- **H1:** Students with higher knowledge of self-medication are more likely to practice it compared to those with less knowledge.
- **H2:** Students with higher knowledge of Adverse Drug Reactions (ADR) are more likely to practice self-medication compared to those with less knowledge.
- **H3:** Students with a positive attitude towards self-medication are more likely to practice it than their counterparts.
- **H4:** Students with a positive attitude towards Adverse Drug Reaction (ADR) are more likely to practice it than their counterparts.
- **H5:** Students who find self-medication more convenient are more likely to practice self-medication than others.
- **H6:** Students who find prescribed medication more inconvenient are more likely to practice self-medication than others

## Methods and materials

### Study design and source of data

This cross-sectional descriptive study utilized primary data, collected from March 21 to April 29, 2023 using a structured questionnaire. This research incorporates quantitative method to analyse data using various statistical modelling techniques such as, descriptive statistics, one-way ANOVA, confirmatory factor analysis (CFA) and structural equation modelling (SEM). The data was obtained through face-to-face interview techniques.

Study area and population. This study includes students from five public universities in Bangladesh. These universities are located in different regions of Bangladesh, and their students come from diverse backgrounds and cultures. Those universities have approximately 78,000 students in undergraduate and graduation program (Supplementary Table 1). This study includes both undergraduate and graduate students. The inclusion criteria are that the person needs to be^1^ an enrolled student from one of the selected universities,^2^ currently continuing their study.^3^ Is willing to participate in the data collection process. Any student who is physically incapable of providing the required information is excluded from this study.

### Questionnaire development

Researchers used a structured five-part questionnaire where each question was considered vital for the research. Questionnaires were first translated into Bangla and then again transformed into English to maintain meaning and consistency. Researchers translated the questionnaire to ask the questions to the participants. As suggested by previous studies, the questionnaires were pre-tested within Khulna University with 30 students before finalizing them for data acquisition^52,53^.

### Dependent variable

This study's dependent variable comprises three parts, each indicating critical aspects of self-medication practice. The first part (Code: PR-1) includes a question about the problem for which the respondent takes self-medication. The problems are divided into three body sections: head, main body, and leg area, with 12 problems for each section which are more prevalent in developing nations^54,55^. Respondents choose the problems for which they take self-medication, and the researcher sums up all the reasons, giving a range of 0–36, with a higher number indicating a greater self-medication practice (Supplementary Table 2).

The second part (**Code: PR-2**) includes a self-medication practice scale comprising 8 items from existing literatures^47,55^**.** and experts’ opinion with a 5-point Likert scale (Supplementary Table 3). The questions are structured positively with no reverse questions and internal reliability (Cronbach’s α = 0.912) and CFA indicates (CMIN/df = 3.986, RMSEA = 0.043, CFI = 0.952, TLI = 0.961) a good fit to consider these 8 questions as a single construct according to Hu and Bentler^56^. Researchers sum up the end result within a range of 8 to 40, with a higher score indicating a higher level of self-medication practice.

The third part (**Code: PR-3**) includes questions about the drugs used for self-medication, with 10 different medicine groups most frequently used worldwide^57,58^ (Supplementary Table 4). The researcher incorporated a 5-point Likert scale to measure the practice of consuming those medicines without a prescription. The reliability among these eight items was within the recommended level (Cronbach’s α = 0.885) along with CFA displayed an overall good fit (CMIN/df = 4.315, RMSEA = 0.049, CFI = 0.963, TLI = 0.986). The final score of this scale is also counted by summing up all the individual questions ranging from 10 to 50, with a higher score indicating a greater level of self-medication practice.

### Independent variables

The study examines the holistic model of self-medication among varsity students by considering four independent variables: knowledge, attitude, convenience of self-medication, and inconvenience of prescribed medication. Each independent variable is assessed using multiple questions presented with a 5-point Likert scale showed in the Table 1. For the Analysis of Variance, the researcher categorizes each construct into three ordinal categories based on the total score of that construct.

**Table 1:** Construct of independent variables.

	Variables	Codes	Groups	Cronbach’s α
Knowledge about medication		Kn-1-1	Primary^6–14^, Moderate (15–22), Advanced (23–30)	
	(a) Content		Not at all, A little, Some, Quite a bit, Very much	0.842
	(b) Doze			
	(c) Regimen (Time)			
	(d) Duration of therapy			
	(e) Precautions			
	(f) Effectiveness			
Knowledge about medicine group		Kn-1-2	Primary (10–25), Moderate (26–40), Advanced (41–50)	
	(a) **Painkillers (**Ex: Diclofenac, Aspirin)		Not at all, A little, Some, Quite a bit, Very much	0.891
	(b) **Anti-acids** (Ex: Maxpro, Sergel, Seclo)			
	(c) **Paracetamol** (Ex: Napa, Ace plus)			
	(d) **Sleeping pills** (Ex: Rivotril, Milam)			
	(e) **Antibiotics** (Ex: Gmax, Amodis, Flazil)			
	(f) **Vitamins** (Ex: Dextram gold, E-cap)			
	(g) **Anti-allergies** (Ex: Encilor, Rupa)			
	(h) **Cough-syrup** (Ex: Dexpoten, Bashok)			
	(i) **Herbal** (Ex: Sinkara)			
	(j) **Anti-Migraine** (Ex: Tafnil, Pyrinol)			
Knowledge about ADR		KN-2	Primary (6–14), Moderate (15–22), Advanced (23–30)	
	Knowledge about Adverse Drug Reactions (ADR)	Kn-2-1	Not at all, A little, Some, Quite a bit, Very much	0.821
	Knowledge about the difference between Side Effects and Adverse Events related to drugs	Kn-2-2		
	Knowledge about the cause of ADR	Kn-2-3		
	Knowledge about buying and consuming medicines without a prescription	Kn-2-4		
	Knowledge about ADR due to not completing or missing the timing of medicine dose	Kn-2-5		
Attitude towards self-medication		AT-1	Positive (6–17), Undecided (18–23), Negative (24–36)	
	Self-medication is not acceptable	AT-1-1	Strongly disagree, Disagree, Unsure, Agree, Strongly agree	0.892
	Students do not have ability to treat symptoms	AT-1-2		
	Self-medication would be harmful	AT-1-3		
	Medical license is essential for medication	AT-1-4		
	Pharmacist is a better source of advice	AT-1-5		
	We should be careful with non-prescribed medicine	AT-1-6		
Attitude towards ADR		AT-2	Positive (4–9), Undecided (10–14), Negative (15–20)	
	ADR reporting by the public is necessary	AT-2-1	Strongly disagree, Disagree, Unsure, Agree, Strongly agree	0.896
	People made aware about ADR reporting	AT-2-2		
	Attend a pharmacovigilance awareness program	AT-2-3		
	Concerned about counterfeit medicines	AT-2-4		
Physical convenience of self-medication		CN-1	Slightly (5–11), Moderately (12–18) Highly (19–25)	
	Buy and store medicine without prescription is easy	CN-1-1	Not at all, A little, Some, Quite a bit, Very much	0.878
	Google can provide necessary information	CN-1-2		
	Previous prescription can suggest	CN-1-3		
	Self-medication is very time saving	CN-1-4		
	Self-medication gives quick relief	CN-1-5		
Physical inconvenience of prescribed-medication		CN-2	Slightly (5–11), Moderately (12–18), Highly (19–25)	
	Health care centers are in a distant position	CN-2-1	Not at all, A little, Some, Quite a bit, Very much	0.825
	Pharmacies are not always readily available	CN-2-2		
	Pharmacy drug provider have limited knowledge	CN-2-3		
	Cost of private medical or doctors is very high	CN-2-4		
	Using health call centers is not convenient	CN-2-5		

### Existing knowledge

Respondents' knowledge was assessed across two dimensions: knowledge of medication and medicine groups, and knowledge about adverse drug reactions (ADR). For knowledge about medication, a 6-item scale adapted from Romero‐Sanchez et al.^59^ was utilized. The scale covered aspects such as dose, content, regimen, and duration. The reliability of these six items was high (Cronbach’s α = 0.842), and confirmatory factor analysis (CFA) indicated a good overall fit (CMIN/df = 3.415, RMSEA = 0.041, CFI = 0.993, TLI = 0.931), aligning with the recommendations of Hu and Bentler^56^.

Similarly, knowledge about the eight most commonly consumed medicine groups^57,58^ was assessed using a five-point Likert scale score ranging from 10 to 40. The reliability among these eight items was within the recommended level (Cronbach’s α = 0.891) along with CFA displayed an overall good fit (CMIN/df = 5.535, RMSEA = 0.052, CFI = 0.982, TLI = 0.954). Knowledge about adverse drug reactions (ADR) was primarily assessed using eight items extracted from previous research^60–62^ However, only five items were found to load into a single item with a factor loading score exceeding 0.5. Therefore, these five items were considered for the final analysis, yielding good fit in Cronbach’s α test (0.821) and in CFA (CMIN/df = 3.821, RMSEA = 0.039, CFI = 0.992, TLI = 0.965).

### Attitude

Attitudes were assessed in two domains: attitude towards self-medication and attitude towards adverse drug reactions (ADR). Attitude towards self-medication was evaluated using 8 items from existing literature^2,29^. Among these, 6 items exhibited factor loading scores above 0.5 and demonstrated high reliability (Cronbach’s α = 0.892). Confirmatory factor analysis (CFA) yielded satisfactory results, indicating a single construct (CMIN/df = 3.427, RMSEA = 0.037, CFI = 0.952, TLI = 0.974). Similarly, attitude towards ADR was assessed with 4 items from previous research^7,29^. These items showed excellent reliability (Cronbach’s α = 0.896), and CFA supported their consideration as a single construct (CMIN/df = 4.215, RMSEA = 0.042, CFI = 0.948, TLI = 0.996).

### Convenience

Convenience was examined from two perspectives: perceived convenience of self-medication and perceived inconvenience of prescribed medication. For perceived convenience of self-medication, 8 items were extracted, specifically tailored for the context of Bangladesh or similar cultural settings^13,63^ Similarly, 6 items were identified for measuring inconvenience of prescribed medication^13,64^.

In both cases, 5 items loaded onto a single component with loading scores exceeding 0.5. Reliability was established with Cronbach’s α scores of 0.878 for convenience of self-medication and 0.825 for inconvenience of prescribed medication. CFA scores for both constructs met recommended values (Convenience of Self-Medication: CMIN/df = 3.255, RMSEA = 0.049, CFI = 0.932, TLI = 0.982; Inconvenience of Prescribed Medication: CMIN/df = 4.659, RMSEA = 0.043, CFI = 0.948, TLI = 0.975) according to Hu and Bentler^56^.

### Sample size determination

The cross-sectional survey was conducted from March 21 to April 29, 2023, utilizing Taro^65^ technique to determine the sample size. Considering a 10% non-response rate, 436 respondents were selected from the total (78,000) population from different universities (Supplementary File Table 1), with a 98% confidence level and a 5% margin of error. Eventually, after sorting all the incomplete and errors datasets, responses from 417 respondents were collected and included for analysis. Calculation process is given below.

$${\text{Taro Yamane Formula}}: {\text{n }} = \frac{N}{{1 + Ne^{2} }}$$

where n, sample size; N, population size; e, level of significance.

$$\begin{aligned} {\text{So}},{\text{ n}} & = \frac{78000}{{1 + 78000*0.05^{2} }} \\ & = \frac{78000}{{1 + 78000*0.0025}} \\ & = \frac{78000}{{1 + 195}} \\ & = \frac{78000}{{196}} \\ \end{aligned}$$

$$\begin{aligned} {\text{n}} & \, = { 397}.{95 }\sim { 398} \\ & = {398} + {1}0\% {\text{ non - response rate}} \\ & = {436} \\ \end{aligned}$$

In SEM, sample size holds particular importance. According to Hair Jr. et al.^66^, a widely recognized guideline suggests that the minimum sample size should be at least 10 times the maximum number of arrowheads pointing at a latent variable within the SEM model. Expanding upon this, Hair Jr. et al.^67^ further elucidated that for Partial Least Squares Structural Equation Modeling (PLS-SEM), considering minimum path coefficients ranging from 0.02 to 0.11, with 0.16 being the lowest path coefficient in our specific model, a sample size exceeding 251 is recommended for a significance level of 1%. Notable, the sample size obtained in our study surpasses this threshold, indicating adequacy for model development.

### Data collection procedure

The data collection technique employed in this study was a random walk, which was the most suitable technique for clusters based on previous research^68,69^. A team of ten trained members carried out the survey. Before data collection, the group underwent a full-day training session covering the data collection procedure, ethics, and obtaining consent. The final sample consisted of 436 participants, and it was divided proportionately among five selected universities based on their student populations. Upon approaching each university, the data collection team divided the campus into blocks and initiated data collection from the main entrance. They proceeded clockwise for one minute and approached the nearest respondent, who fulfilled all the inclusion criteria. This process was repeated until the predetermined quota was achieved. In cases where a selected respondent declined to cooperate, the team labeled it a non-response and continued with the data collection process. The same technique was applied consistently throughout the entire data collection phase.

### Statistical analysis

During the data curation process, the interviewer reported a non-response rate of approximately 3%. Additionally, 8 respondents were disregarded due to missing or improper data, resulting in 417 data sets being considered for analysis. The data were stored, coded with appropriate weights, and categorized based on knowledge, attitude, convenience, and practice segments. Descriptive analysis was performed to present the data, followed by one-way ANOVA with post-hoc Least Significant Difference (LSD) to identify any mean differences among groups, showing which group used more self-medication. Finally, a Structural Equation Model (SEM) was developed to assess the causal relationship between self-medication practice with knowledge, attitude, and convenience. SEM was chosen over linear regression due to its ability to accommodate multiple dependent variables, as in this case where three distinct constructs are involved. Additionally, SEM enables the depiction of latent variables and the examination of both direct and indirect effects, making it a powerful tool for testing theoretical models^70^. The internal consistency of the questions for each section was assessed using Composite reliability and Average variance extracted (AVE). The results for every scale used were within the recommended levels (Composite reliability > 0.80 and AVE > 0.50).

### Ethics approval

This study was carried out in order to partially fulfillment of the Master’s degree in Development Studies (MDS) from Khulna University, Bangladesh. The discipline and supervisor of this study protocol was approved. In addition, Ethical Clearance Committee of Research and Innovation Centre (RIC), Khulna University, Bangladesh has been approved this whole study protocol. The reference number is (KUECC-2023–09-61). All the methods are in accordance with relevant guidelines and regulations.

### Consent for publication

Informed consent was obtained from all the participants.

## Results

Table 2 presents the demographic characteristics of the respondents. Most participants are male, accounting for 64% of the sample. No significant differences were observed between males and females regarding self-medication in any dimension. The CGPA of the respondents exhibited a significant difference in the real-life practice of self-medication. Individuals with a low CGPA had a higher mean score for self-medication (mean RP: 23.45; P = 0.003). However, there were no differences in drug usage or disease treatment associated with self-medication among different CGPA groups. The year of study did not have any effect on the self-medication practices of the students. Regardless of their academic year, all students demonstrated similar patterns of self-medication. The study area was found to have a significant effect on self-medication practices. Surprisingly, students studying pure science or applied science reported significantly higher self-medication levels than other groups. They treated more diseases with self-medication, used a greater variety of drugs, and had higher overall self-medication scores. Science students are assumed to have more knowledge about medication and its potential adverse effects, yet they still engage in self-medication. The convenience of self-medication over prescribed medication may play a significant role in this regard. Economic dependence did not significantly affect self-medication practices among the respondents.

**Table 2:** Demographic profile of the respondents.

	Variables	Frequency (Percentage)	Diseases treated (DT)		Real life practice (RP)		Drug used (DU)	
			Mean	Sig.	Mean	Sig.	Mean	Sig.
Sex of the respondents								
	Male	265 (64)	14.22	0.241	22.11	0.092	28.19	0.117
	Female	152 (36)	14.89		21.78		29.31	
	CGPA							
	Below 2.00	32 (7.03)	12.47	0.121	23.45	0.003	27.81	0.096
	2.01–3.00	119 (28.89)	13.15		21.88		27.56	
	3.01–3.50	172 (41.95)	14.24		22.91		28.90	
	3.51–4.00	89 (22.11)	12.27		20.97		29.23	
Year of study								
	Undergraduate	238 (57.28)	14.01	0.335	23.14	0.271	28.67	0.421
	Master’s program	136 (31.90)	13.66		22.56		28.10	
	PhD or other	43 (10.80)	13.80		21.92		29.59	
Study area								
	Pure science	94 (22.36)	16.13	0.002	25.34	< 0.001	30.14	0.019
	Applied science	137 (30.90)	15.76		26.11		29.35	
	Social Science	98 (22.86)	13.60		20.25		26.18	
	Arts	44 (9.54)	12.92		21.67		25.74	
	Business	57 (14.32)	14.22		19.92		28.02	
Economic dependence								
	Dependent on others	261(62.58)	14.01	0.248	24.12	0.345	27.58	0.076
	Self-dependent	156 (37.41)	14.92		23.87		28.79	

Table 3 presents those individuals with intermediate knowledge about medication (mean DT: 17.65) who diagnose significantly more diseases with self-medication than other groups. Post hoc LSD results show that they treat more diseases than those with higher medication knowledge (mean DT: 15.60). Similarly, those with intermediate medication knowledge (mean RP: 23.57) have the highest level of self-medication practice, even more than those with higher medication knowledge (mean RP: 21.86). However, those with intermediate knowledge of medication do not use a variety of medicine groups for treating many diseases. Table 3 shows that those with the highest knowledge about medication (mean 7) use a variety of medicine groups in self-medication. Post hoc LSD results reveal a positive relationship between knowledge about medication and variety of medicine intake.

**Table 3:** One way ANOVA test results.

	Variables	Frequency	Diseases treated (DT)			Real life practice (RP)			Drug used (DU)		
			Mean	SD	Sig.	Mean	SD	Sig.	Mean	SD	Sig.
Knowledge about medication											
	Primary	104	13.25	3.346	< 0.001	17.56	6.026	< 0.001	24.40	5.263	< 0.001
	Intermediate	233	17.65	3.456		23.57	5.224		29.07	6.066	
	Advance	80	15.60	4.815		21.86	8.327		31.61	8.662	
Knowledge about medicine groups											
	Primary	126	13.92	3.722	< 0.001	18.31	5.971	< 0.001	26.20	5.888	< 0.001
	Intermediate	209	16.11	3.371		22.01	5.846		29.28	6.702	
	Advance	82	19.71	4.185		26.32	6.444		33.26	7.353	
Knowledge about adverse drug reaction											
	Primary	199	13.92	3.620	< 0.001	19.41	6.478	0.005	26.56	7.096	0.002
	Intermediate	181	17.66	3.432		23.23	6.027		31.19	6.282	
	Advance	37	20.84	2.609		27.00	4.679		32.89	4.881	
Attitude towards self-medication											
	Positive	197	18.39	3.274	0.011	26.39	4.066	< 0.001	32.51	5.881	< 0.001
	Undecided	86	15.92	3.312		20.42	5.157		26.69	6.774	
	Negative	134	13.04	3.736		15.76	5.095		25.73	6.460	
Attitude towards adverse effect of self-medication											
	Positive	167	18.72	3.158	0.007	26.34	4.574	< 0.001	32.35	5.979	< 0.001
	Undecided	104	16.47	3.050		21.44	4.784		29.96	6.729	
	Negative	146	13.01	3.722		16.69	5.901		24.86	6.144	
Physical convenience of self-medicating											
	Slightly	70	13.03	4.177	< 0.001	16.19	6.493	0.003	23.21	6.972	0.015
	Moderately	101	14.41	3.761		19.44	6.185		25.50	6.612	
	Highly	246	17.77	3.456		24.27	5.400		32.30	5.176	
Physical inconvenience of prescribed-medication											
	Slightly	101	13.61	4.108	< 0.001	18.26	6.580	< 0.001	22.18	6.966	< 0.001
	Moderately	108	16.12	3.642		21.00	5.701		27.46	4.850	
	Highly	208	17.41	3.863		23.82	6.277		33.37	4.530	
**Source:** Field survey 2023											

Knowledge about different medicine groups individuals with higher knowledge about other medicine groups (mean DT: 19.71) treat more diseases with self-medication. Their mean score is significantly higher than the second-highest group, indicating the impact of knowledge on self-medication effectiveness. Individuals with a positive attitude towards self-medication practice it more frequently (mean RP: 26.39), diagnose more diseases (mean DT: 18.39), and take different medicine (mean DU: 32.51). Those with a negative attitude or uncertainty about self-medication show no significant differences in practice scales. Individuals with a negative attitude towards adverse drug reactions have lower scores in all practice scales (mean DT: 13.01, mean DU: 24.86, mean RP: 16.69). Those with a positive attitude practice more self-medication and take a greater variety of medicines.

Respondents who find self-medication highly convenient or prescribed medication highly inconvenient practice self-medication more frequently. Physical convenience or inconvenience plays a significant role in shaping the practice of self-medication. Those who find prescribed medication slightly inconvenient (mean DT: 13.61, mean RP: 18.26, mean DU: 22.28) score significantly lower in all self-medication practice scales. The physical inconvenience of prescribed medication plays a more significant role in pushing people towards self-medication than the convenience of self-medication. Challenges associated with obtaining prescribed medication contribute to the prevalence of self-medication in Bangladesh.

### Measurement model (Model fit indices)

**Figure 2:**
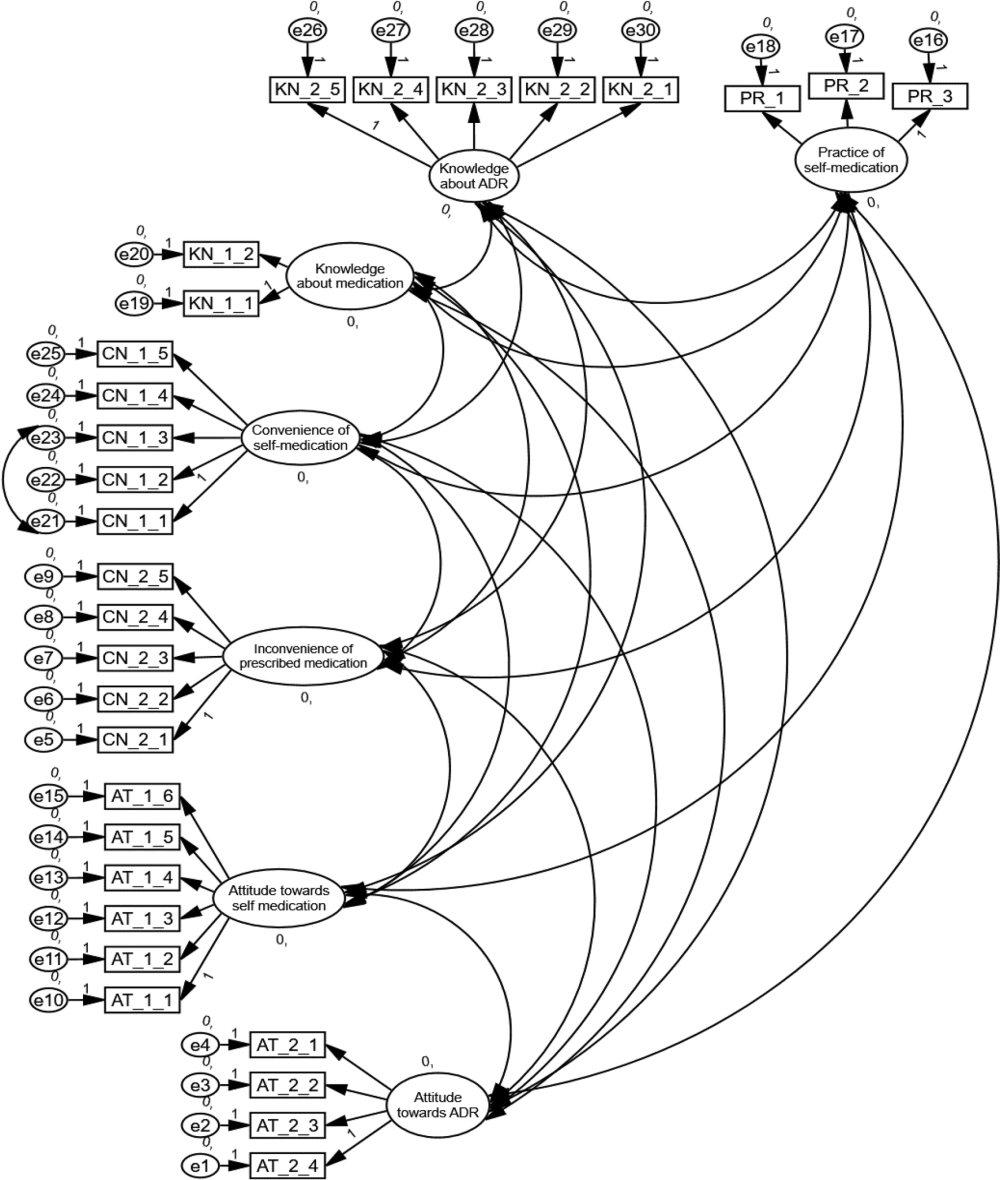
Confirmatory factor analysis.

Confirmatory factor analysis (CFA) was performed using AMOS. 26 to run the SEM model (Fig. 2). All the factors with loading scores (> 0.50) were considered fit for the model. This model is well validated and fit for SEM computation because all the values are under an acceptable threshold. The CMIN/df = 5.023; CFI = 0.902; GFI = 0.911; TLI = 0.901; RMSEA = 0.073 and SRMR = 0.052 (Supplementary Table 5).

### Composite reliability (CR) and Average Variance (AVE) test

In this case, the composite reliability scores ranged from 0.838 to 0.932, which is well above the benchmark of 0.70, indicating that the items used to measure the constructs have high reliability. In this study, the AVE values for all the constructs were above 0.50, indicating that the items used to measure each construct are strongly related to that construct (Table 4). While the (Supplementary Table 4) portrays that a value of Cronbach’s α (0.885) indicates high internal consistency among the survey items. It suggests that the items in our questionnaire are closely related and measure a single, reliable construct. While (CMIN/df = 4.315) this is the ratio of the chi-square statistic to degrees of freedom in Confirmatory Factor Analysis (CFA). A lower value suggests a better fit, and a value around 4 is generally considered acceptable whereas the Root Mean Square Error of Approximation (RMSEA) measures the discrepancy between the model and the observed data. A value of (0.049) in our study indicates a good fit, as values below 0.05 are often considered excellent. Other measurements of Comparative Fit Index (CFI = 0.963) measures how well the model fits the data compared to a null model. A value of 0.963 is generally considered good, with higher values indicating a better fit. Tucker-Lewis Index (TLI = 0.986) is another fit index. A value of 0.986 indicates a high level of fit, with values close to 1 indicating a good fit.

**Table 4:** Composite reliability test analysis results.

	Variables (codes)	Factor loading	Composite reliability	AVE
Knowledge about medication (KN-1)				
	KN-1-1	0.644	0.838	0.732
	KN-1-2	0.924		
Knowledge about the adverse effect of drug reaction (KN-2)				
	KN-2-1	0.793	0.919	0.696
	KN-2-2	0.838		
	KN-2-3	0.851		
	KN-2-4	0.826		
	KN-2-5	0.861		
Attitude towards self-medication (AT-1)				
	AT-1-1	0.920	0.932	0.769
	AT-1-2	0.852		
	AT-1-3	0.921		
	AT-1-4	0.828		
	AT-1-5	0.858		
	AT-1-6	0.879		
Attitude towards adverse effect of drug reaction (AT-2)				
	AT-2-1	0.800	0.897	0.686
	AT-2-2	0.796		
	AT-2-3	0.838		
	AT-2-4	0.875		
Physical convenience of self-medication (CN-1)				
	CN-1-1	0.806	0.915	0.684
	CN-1-2	0.860		
	CN-1-3	0.786		
	CN-1-4	0.840		
	CN-1-5	0.841		
Physical inconvenience of prescribed medication (CN-2)				
	CN-2-1	0.863	0.926	0.777
	CN-2-2	0.901		
	CN-2-3	0.893		
	CN-2-4	0.881		
	CN-2-5	0.869		
Self-medication practice (PR)				
	PR-1	0.750	0.849	0.653
	PR-2	0.852		
	PR-3	0.819		
*Source: Field Survey 2023				

### Discriminant validity test

Two criteria were utilized to assess the discriminant validity of the measurement model: Fornell and Larcker’s criterion and the Heterotrait-Monotrait (HTMT) ratio. The outcomes of the Fornell and Larcker criterion, displayed in supplementary table 6, reveal that the square root of the AVE for each construct is higher than its correlation with other constructs. This signifies that discriminant validity has been established for all the constructs. All the HTMT ratios were below 0.85, ranging from 0.273 to 0.762 (Supplementary Table 7), which indicates that discriminant validity is established for the measurement model according to the HTMT ratio criterion while the Fornell and Larcker criterion is a method for assessing discriminant validity in a measurement model. Discriminant validity ensures that the measures used to assess different constructs are distinct and not measuring the same underlying concept. In the (Supplementary Table 6) where PR: AVE = 0.808 (Diagonal element) while the discriminant validity is supported if the correlations with other constructs (off-diagonal elements) are smaller than 0.808. The correlation with KN-2 is 0.607**, which is smaller than 0.808, supporting discriminant validity whereas KN-2 (Knowledge-2): AVE = 0.834 (Diagonal element). Discriminant validity is supported if the correlations with other constructs (off-diagonal elements) are smaller than 0.834. The correlation with PR is 0.607**, which is smaller than 0.834, supporting discriminant validity.

### Structural equation model (SEM)

The researcher initially developed a hypothesized structural model to investigate self-medication practice. However, upon evaluating the modification indices and parameter estimates, some paths in the model were identified as non-significant. As a result, the researcher removed those non-significant paths and added several covariance paths, as suggested by the modification indices. The final model showed a better fit compared to the hypothesized model.

**Figure 3:**
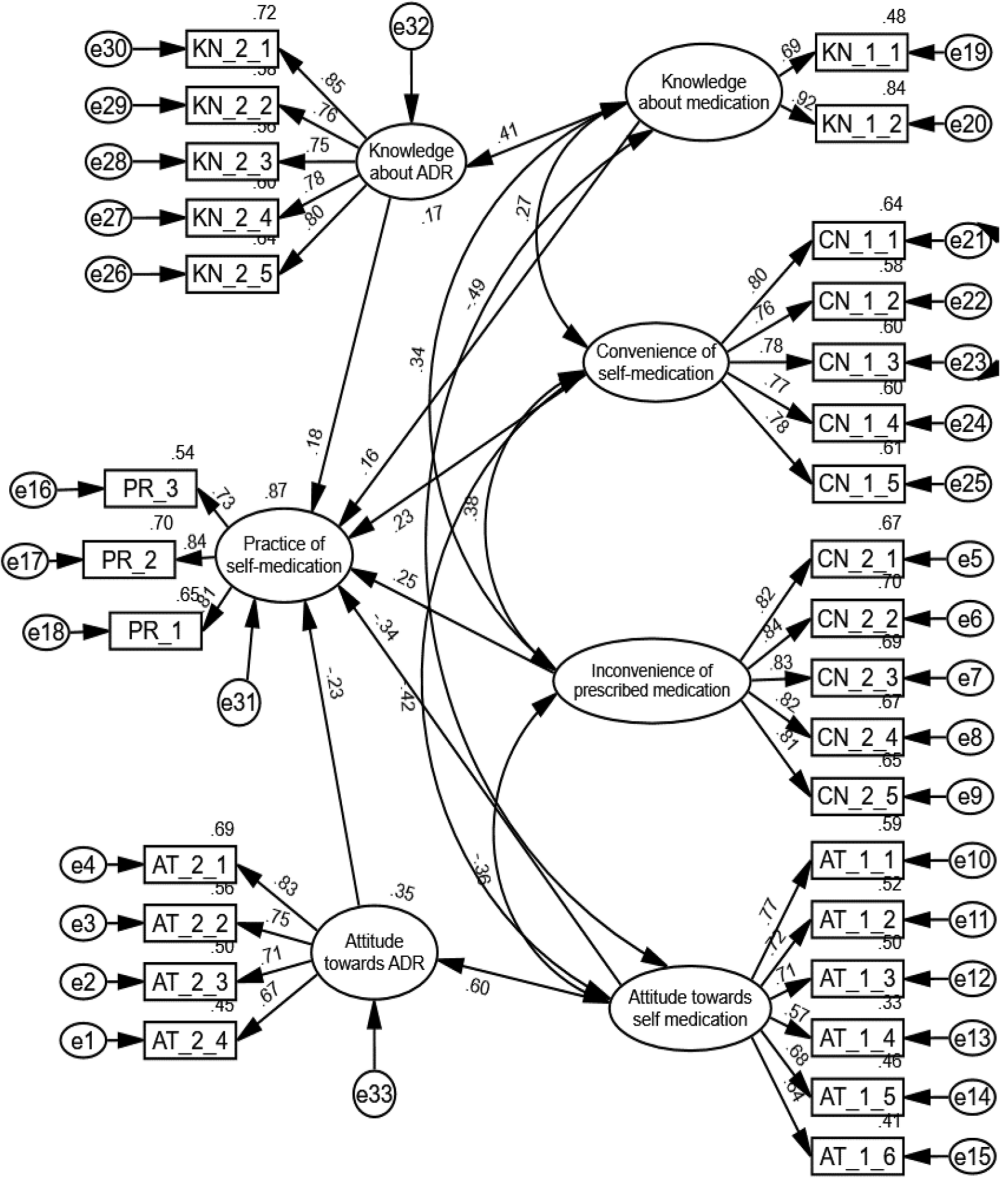
Structured equation model for self-medication practice.

In the paper, six hypotheses were presented regarding self-medication practice, all supported by the presented Structural Equation Model (Fig. 3). To assess the model's fit, Table 5 is presented, which displays the model’s fit and estimates for the constructs. The table shows that the model is acceptable and is a good fit for the data, as indicated by the following fit indices: CMIN/df = 4.728, CFI = 0.923, GFI = 0.916, RMSEA = 0.070, and SRMR = 0.0511. These findings suggest that the model is reliable and valid for investigating self-medication practices.

**Table 5:** Hypothesis testing results for structural equation model (SEM).

Hypothesis (Relationships)	β	t-value	p-value	Hypothesis results	SDE	SIE	STE
KN-1 → PR	0.160	3.610	< 0.001	Accepted	0.160	0.072	0.232
KN-2 → PR	0.176	4.773	< 0.001	Accepted	0.176	0	0.176
AT-1 → PR	− 0.340	− 6.066	< 0.001	Accepted	− 0.340	− 0.140	− 0.480
AT-2 → PR	− 0.235	− 5.041	< 0.001	Accepted	− 0.235	0	− 0.235
CN-1 → PR	0.235	5.987	< 0.001	Accepted	0.235	0	0.235
CN-2 → PR	0.252	6.529	< 0.001	Accepted	0.252	0	0.252
Fit indexes: CMIN/df = 4.728; CFI = 0.923; GFI = 0.916; RMSEA = 0.070; SRMR = 0.0511							

SDE, standardized direct effect; SIE, standardized indirect effect; STE, standardized total effect.

The final measurement model found that attitude towards self-medication had the highest significant effect (β = − 0.340; t = − 6.066) on self-medication practice, indicating a negative correlation. Those with a negative attitude towards self-medication were found to practice self-medication less. Table 5 further demonstrated that attitude towards self-medication had the highest standardized direct (β = − 0.340; p =  < 0.001) and indirect effect (β = − 0.140; p = 0.005) on self-medication practice, suggesting that individuals who view self-medication as unacceptable tend to practice it less compared to others.

Similarly, a negative attitude towards adverse drug reactions (β = − 0.235; t = − 5.041) was found to have a significant negative effect on self-medication practice. The physical inconvenience of prescribed medication had the second highest effect (β = 0.252; t = 6.529) on self-medication practice, indicating that those who find the prescribed medication process inconvenient in Bangladesh tend to practice self-medication more. Physical convenience of self-medication had the third largest effect (β = 0.235; t = 6.529) on self-medication, indicating that the convenience of self-medication is one of the main factors behind the high prevalence of self-medication in Bangladesh.

Furthermore, knowledge about medication (β = 0.160; t = 3.610) and adverse drug reactions (β = 0.176; t = 4.773) had significant positive effects on self-medication practice, which was surprising as it was expected that individuals with greater knowledge about medication and its adverse effects would be less likely to self-medicate.

## Discussion

The study's findings indicate a moderate to a high level of self-medication practice among university students in Bangladesh, consistent with previous studies^35,36,38,72,73^. Several factors contribute to this practice, including limited access to healthcare services, cost-effectiveness, cultural beliefs, and the convenience of purchasing medications without a prescription from pharmacies and drug stores^74^.

The researcher focused on two types of knowledge among university students in Bangladesh: knowledge about medication and knowledge about adverse drug reactions (ADRs). Knowledge about medication was assessed based on medication doses, content, and related aspects, as well as knowledge of different medicine groups. The findings revealed that most students possessed intermediate knowledge about medication, with a relatively higher level of knowledge about medicine groups, consistent with previous studies conducted in different cities^27^. This observation could be attributed to the country's heavy marketing efforts of pharmaceutical companies. Surprisingly, most students exhibited only basic knowledge of ADRs. Previous research indicated that students often hold misconceptions, believing that non-severe illnesses cannot lead to serious consequences^29^. This knowledge gap regarding the risks associated with self-medication might be one of the reasons for the high prevalence of self-medication among university students in Bangladesh. The limited awareness about ADRs among students could be attributed to the insufficient emphasis on pharmacovigilance in the country.

The structural equation model (SEM) presented in Fig. 1 reveals a positive causal relationship between knowledge about self-medication and adverse drug reactions (ADRs) and the practice of self-medication. Previous research^75–77^ supports this by indicating that individuals who engage in self-medication within the last 12 months tend to have a higher level of knowledge about self-medication. Furthermore, the SEM suggests that knowledge about medication indirectly affects self-medication by influencing knowledge about ADRs. Thus, those with greater knowledge about self-medication are likely to possess more knowledge about ADRs and higher self-medication practice. The belief that self-medication is an effective and convenient way to manage minor health problems, coupled with university students’ confidence in their medication knowledge^78^, may play a role. Additionally, in Bangladesh, the easy availability of over-the-counter medicine and the convenience of storing it^78,79^ make self-medication a preferred choice. Conversely, obtaining prescribed medication is more difficult and inconvenient^78^, as observed in this study. Consequently, knowledgeable individuals may opt for the convenient, easily accessible, and quick relief self-medication provides.

Contrasting findings were presented by a study conducted in Portugal^41^ found no positive connection between knowledge and the practice of self-medication. Similarly, a study in Nepal^80^ revealed that individuals with higher knowledge about medication safety were less likely to engage in self-medication. Additionally, a study in Nigeria^75^ showed a negative correlation between knowledge about ADRs and self-medication practices among university students. These findings highlight the complex nature of the relationship between knowledge and self-medication, suggesting that various factors influence it. While university students in Bangladesh continue to engage in self-medication despite understanding the associated risks, others may avoid it due to their awareness of potential consequences. The results of this study indicate that most university students in the country have a positive attitude towards self-medication and adverse drug reactions (ADRs). Previous studies on general university and medical students have also shown that most of them hold a positive attitude toward self-medication^17,29,32,73,79,81^. However, some studies suggest that students generally have a negative attitude toward self-medication^27,81,82^ and are cautious about recommending self-medication to friends and family members^83^. These studies further suggest that students may develop this mindset due to increased awareness about medications and ADRs during their time in university^28,27^.

Moonajilin et al.^13^ found that, on average, only 26.3% of Bangladeshi people seek healthcare facilities for treatment, indicating a significant shortage in healthcare infrastructure. This shortage could be a prominent reason for the positive attitude towards self-medication and ADRs. Additionally, cultural and traditional beliefs have been identified as influential factors in shaping attitudes towards self-medication^74,84^, and the cultural and traditional beliefs in Bangladesh may have played a significant role in this context. Moreover, the present study has shown that students have limited knowledge about ADRs and their potential risks, which could contribute to these unique findings. This study reinforces the consistent finding from previous studies^28,41,73^ that individuals with a positive attitude towards self-medication and adverse drug reactions (ADRs) tend to engage in self-medication more frequently. In contrast, those with a negative attitude are less inclined to do so. Among university students, except for some instances presented by James^28^, a positive attitude towards self-medication is a common factor^78,85^. The SEM (Fig. 1) used in this study also confirms that a positive attitude towards self-medication has the strongest influence on the actual practice (β = − 0.34; p-value < 0.001) encouraging the use of various medicine groups, treating different diseases through self-medication, and increasing the frequency of self-medication.

Furthermore, the study reveals that the attitude toward self-medication indirectly impacts the practice by influencing the attitude toward adverse drug reactions (ADRs). This suggests that individuals who perceive self-medication as acceptable also subconsciously acknowledge the potential negative consequences, despite recognizing its effectiveness. It can be attributed to self-medication as a convenient and cost-effective option to get quick relief from minor health issues^41,74^. Additionally, from a psychological perspective, one can argue that university students, being mature and knowledgeable individuals, possess optimism while accepting the reality of self-medication. This acknowledgment demonstrates their understanding that their choices can have both positive and negative outcomes, and they are willing to accept the responsibility that comes with it.

This study highlights the widespread perception among students that self-medication is highly convenient. They report that buying and storing medicine in Bangladesh is easy, consistent with previous studies^48,29,63^ conducted in Bangladesh and other countries^83,86,87^. Those over-the-counter (OTC) medications are commonly used for self-medication. Students also emphasize that self-medication saves time and offers quick relief, which has consistently emerged as primary motivation for self-medication in previous studies^7,88,89^. Additionally, the convenience of self-medication in Bangladesh is attributed to the knowledge gained from previous prescriptions and the online availability of medical information. These factors have also been reported as key drivers of self-medication^78,81,90^. The structural equation model (SEM) shows a significant positive causal relationship between the convenience of self-medication (β = 0.23; p-value < 0.001) and its practice. As the convenience of self-medication increases, so does its prevalence. This finding is consistent with earlier studies^28,32,34,38,39,71,72^ highlighting the positive influence of easy access to self-medication on its practice.

One possible explanation for the convenience of self-medication is the proliferation of health-related resources on the internet. This enables individuals to independently research symptoms, diagnoses, and potential treatments, leading to a sense of autonomy and confidence in making health decisions without consulting a healthcare professional. Additionally, easy access to medical information allows students to become more knowledgeable about common ailments, symptoms, and treatments, thereby reinforcing the practice of self-medication^78,81,90^. This study highlights the significant inconvenience half of the students face when accessing prescribed medicine in Bangladesh, shedding light on the challenges associated with healthcare services in the country. The reasons identified by the study, including distance to government healthcare centers, unavailability of pharmacies, high cost of private medical care, and inadequate health call centers, are all significant barriers to accessing healthcare services. Previous studies conducted in Bangladesh^90,91^ have also reported difficulties obtaining advice from healthcare professionals, further exacerbating the problem. This situation can be directly attributed to the low healthcare budget in Bangladesh, which is one of the lowest in South Asia, accounting for only 5.4% of the total GDP^14^.

The study reveals a positive causal relationship between the physical inconvenience of obtaining prescribed medication and the practice of self-medication, as indicated by the structural equation model (SEM) analysis (β = 0.25; p-value < 0.001). Consequently, the practice of self-medication among university students increases with the growing inconvenience of accessing prescribed medication. These findings align with previous research^74,78,79,83,84^ that has identified difficulties in obtaining proper treatment as a significant contributing factor to the practice of self-medication. Earlier, this study stated that students with a higher level of medication knowledge resort to self-medication may be due to the significant inconvenience associated with obtaining prescribed medication. This inconvenience can also be attributed to the accepting attitude of students towards the potential negative consequences that may arise due to self-medication. Students in Bangladesh may prefer to avoid the hassle of seeking health professionals' suggestions, which contributes to this phenomenon.

## Strengths and limitations

The strengths and weaknesses of this study have been accredited. The strength that lies in this study is the greater generalizability of the findings since it covered greater participants and universities. Along with appropriate statistical methods and casual modelling are the main strengths of this study. SEM allows researchers to model and test complex relationships among multiple variables simultaneously. This is particularly beneficial when investigating intricate structures or pathways in cross-sectional data. SEM incorporates measurement models, which can help account for measurement errors in observed variables. By doing so, it provides a more accurate estimation of the relationships between latent constructs.

While the limitations are its cross-sectional nature limits the casual inference since outcomes and predictors variables have been collected same time frame. Therefore, SEM allows for the testing of causal relationships, establishing true causality in cross-sectional studies can be difficult. The observed relationships may be influenced by unobserved variables or bidirectional causation. While the ability to model complex relationships is a strength, it can also be a limitation. Overly complex models with too many parameters can lead to overfitting and poor generalization to new data.

## Policy recommendations

Despite existing regulations and healthcare facilities, effective and evidence-based guidelines are needed to improve self-medication practices. While self-medication can be beneficial if appropriately directed, but specific actions are needed.

- To strengthen prescribed medication practices, standardized guidelines should be developed, covering dosage recommendations, regimen instructions, and considerations for potential adverse effects. Healthcare providers should undergo continuous education on evidence-based medicine and patient safety, with mechanisms in place to monitor and evaluate adherence to guidelines.
- Enhancing the quantity and accessibility of prescribed medication requires collaboration between governments, healthcare providers, and pharmaceutical companies. Measures include ensuring an adequate supply of medications, reducing costs through bulk purchasing agreements and promoting generics, and improving accessibility through pharmacy networks, home delivery, and telemedicine.
- Improving government medical services entails increased funding for infrastructure, equipment, and staff capacity. Reducing waiting times and implementing electronic health record systems can enhance patient satisfaction and care coordination.
- Educating the public is vital. Public awareness campaigns should highlight self-medication risks, while collaboration with educational institutions and healthcare providers can deliver targeted programs on proper medication usage. Leveraging communication channels is essential to disseminate accurate information and discourage self-medication.
- Strengthening regulation and enforcement involves monitoring and controlling the availability and sales of over-the-counter medications, deterring unauthorized prescription sales, and raising awareness of legal and health consequences.
- There might be potential differences across different groups of university students in terms of practicing self-medications therefore further detailed study is recommended to explore these differences across different study level.

## Conclusion

In conclusion, university students in Bangladesh possess intermediate medication knowledge and primary knowledge of Adverse Drug Reactions (ADRs). They exhibit a positive attitude towards both self-medication and ADRs. Physical convenience favors self-medication, while the inconvenience of prescribed medication contributes to its lower preference. Students with advanced medication knowledge and positive attitudes toward self-medication are more likely to engage in it. This research is crucial for self-medicating students and policymakers seeking to improve control measures. Further research should explore age-specific factors such as individual beliefs, cultural influences, and socio-economic factors that drive self-medication preferences.

### Supplementary Information

Supplementary Tables.

## Data Availability

The datasets used and/or analysed during the current study available from the corresponding author on reasonable request.
