# Supplementary material for: Knowledge attitude and convenience on self-medication practices among university students in Bangladesh exploration using structural equation modeling approach
Source: Sci Rep. 2024 May 12;14:10837. doi: 10.1038/s41598-024-60931-9 (PMC11089040; doi:10.1038/s41598-024-60931-9)
Supplement: Supplementary file 1 — Supplementary Tables. [file 41598_2024_60931_MOESM1_ESM.docx]

Supplementary Table 1: The approximate number of University Students.

| **Universities** | **Undergraduate** | **Graduate** |
| --- | --- | --- |
| Khulna University | 5000 | 2000 |
| Dhaka University | 23000 | 17000 |
| Barisal University | 2000 | 1000 |
| BSMRSTU | 8000 | 2000 |
| Jahangirnagar University | 12000 | 6000 |
| **Total** | **50000** | **28000** |
| **Source**: ugc-universities.gov.bd (2023) | | |

Supplementary Table 2: List of disease for which one take self‐medication


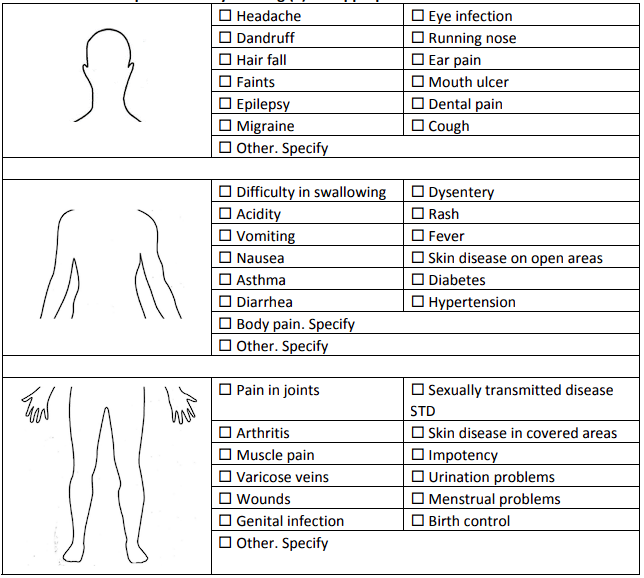


Supplementary Table 3: Self-medication practice scale

| **Variables (Code: PR-2)** | | **Responses** | | | | |
| --- | --- | --- | --- | --- | --- | --- |
|  |  | Never | Rarely | Sometimes | Often | Always |
|  | 1. Do you follow Self-prescription? |  |  |  |  |  |
|  | 2. Do you discontinue the prescribed medicines by yourself when symptoms are not relieved? |  |  |  |  |  |
|  | 3. Do you reuse the prescription when experienced with similar symptoms? |  |  |  |  |  |
|  | 4. Do you increase the drug dose on yourself when symptoms are not relieved? |  |  |  |  |  |
|  | 5. Do you experience positive reactions during self-medication? |  |  |  |  |  |
|  | 6. Are you habitual to any drug? |  |  |  |  |  |
|  | 7. Do you give your prescription to someone who is having similar symptoms as yours before? |  |  |  |  |  |
|  | 8. Do you combine herbal medicine and western medicine? |  |  |  |  |  |
| -Cronbach’s α = 0.912  -CFA Scores: CMIN/df = 3.986, RMSEA = 0.043, CFI = 0.952, TLI = 0.961 | | | | | | |

Supplementary Table 4: Questions about drug used for self-medication

|  | **Code: PR-3** | **Responses** | | | | |
| --- | --- | --- | --- | --- | --- | --- |
|  | Variables | Never | Rarely | Some  times | Often | Always |
| 1 | **Painkillers (**Ex: Diclofenac, Aspirin) |  |  |  |  |  |
| 2 | **Anta-acids** (Ex: Maxpro, Sergel, Seclo) |  |  |  |  |  |
| 3 | **Paracetamol** (Ex: Napa, Ace plus) |  |  |  |  |  |
| 4 | **Sleeping pills** (Ex: Rivotril, Pase, Milam) |  |  |  |  |  |
| 5 | **Antibiotics** (Ex: Gmax, Amodis, Flazil) |  |  |  |  |  |
| 6 | **Vitamins** (Ex: Dextram gold, E-cap, C-vit) |  |  |  |  |  |
| 7 | **Anti-allergies** (Ex: Encilor, Rupa) |  |  |  |  |  |
| 8 | **Cough-syrup** (Ex: Dexpoten, Bashok) |  |  |  |  |  |
| 9 | **Herbal** (Ex: Sinkara) |  |  |  |  |  |
| 10 | **Anti-Migraine** (Ex: Tafnil, Pyrinol) |  |  |  |  |  |
| -Cronbach’s α = 0.885  -CFA scores: CMIN/df = 4.315, RMSEA = 0.049, CFI = 0.963, TLI = 0.986 | | | | | | |

Supplementary Table 5: Model-fit indexes for the SEM model

| **Fit Index** | **Recommended value** | **Obtained value** |
| --- | --- | --- |
| CMIN/df | 3-5 | 5.023 |
| CFI | >.90 | 0.902 |
| GFI | >.90 | 0.911 |
| TLI | >.90 | 0.901 |
| RMSEA | <.08 | 0.073 |
| SRMR | <.08 | 0.052 |

Supplementary Table 6: Fornell and Larcker criterion for discriminant validation

|  | **PR** | **KN-2** | **Kn-1** | **AT-2** | **CN-2** | **CN-1** | **AT-1** |
| --- | --- | --- | --- | --- | --- | --- | --- |
| **PR** | **0.808** |  |  |  |  |  |  |
| **KN-2** | 0.607** | **0.834** |  |  |  |  |  |
| **Kn-1** | 0.549** | 0.354** | **0.855** |  |  |  |  |
| **AT-2** | -0.759** | -0.520** | -0.325** | **0.828** |  |  |  |
| **CN-2** | 0.626** | 0.271** | 0.284** | -0.370** | **0.882** |  |  |
| **CN-1** | 0.677** | 0.400** | 0.219*** | -0.590** | 0.455** | **0.827** |  |
| **AT-1** | -0.780** | -0.487** | -0.425** | 0.663** | -0.326** | -0.412** | **0.877** |

Supplementary Table 7: Heterotrait-Monotrait (HTMT) ratio

|  | **PR** | **KN-2** | **Kn-1** | **AT-2** | **CN-2** | **CN-1** | **AT-1** |
| --- | --- | --- | --- | --- | --- | --- | --- |
| **PR** | **1** |  |  |  |  |  |  |
| **KN-2** | 0.608 | **1** |  |  |  |  |  |
| **Kn-1** | 0.580 | 0.394 | **1** |  |  |  |  |
| **AT-2** | 0.762 | 0.532 | 0.327 | **1** |  |  |  |
| **CN-2** | 0.676 | 0.268 | 0.334 | 0.370 | **1** |  |  |
| **CN-1** | 0.707 | 0.406 | 0.273 | 0.607 | 0.460 | **1** |  |
| **AT-1** | 0.755 | 0.486 | 0.385 | 0.667 | 0.322 | 0.421 | **1** |
